# How employees can break out of their learning comfort zone in green innovation scenarios: a nudging experiment based on the pressures of sustainable development in China

**DOI:** 10.3389/fpsyg.2025.1430125

**Published:** 2025-06-23

**Authors:** Shuming Li, Chuanxi Yang, Guangyu Zhai

**Affiliations:** ^1^School of Economics and Management, Lanzhou University of Technology, Lanzhou, China; ^2^School of Business, Guilin Tourism University, Guilin, China

**Keywords:** green innovation, combination nudge, isolation nudge, green knowledge, sustainable development

## Abstract

The use of nudging tools to motivate employees to actively participate in corporate green innovation has not yet received sufficient attention. Designing and implementing effective nudging strategies to push employees out of their comfort zones and actively learning green knowledge can bridge the research gap based on the expectancy theory. In this study, 2,253 participants from Chinese manufacturing firms were divided into five groups to investigate the effect of different nudges on green knowledge acquisition. The findings indicate that the combined nudges of social norm and social status have a greater impact than their individual counterparts, and there is no evidence of a crowding out effect. It is more meaningful for employee learning and corporate green development that the efficacy of praising before pressuring is greater in the two combined interventions. Furthermore, we revalidated the efficacy of nudging tactics by carrying out robustness tests and heterogeneity verification for subdivided samples of enterprise ownership and employee position. This study offers a viable operational pathway for theoretical research and business practice on green innovation. We are willing to suggest that stakeholders devote more effort to studying various types of innovation nudging methods.

## 1 Introduction

Under the surge of green innovation, cultivating workforce engagement in perpetual skill enhancement and renewing green knowledge constitutes an indispensable strategy for mitigating sustainability dilemmas (Behera and Sethi, 2022; Xavier et al., 2016). However, due to the inherent learning inertia of individuals, employees are prone to remain entrenched in their comfort zones, exhibiting resistance to altering the status quo (Ritala et al., 2015). How to choose tools with high freedom, low cost and high efficiency to motivate employees’ green knowledge learning becomes a significant and important fundamental task (Benartzi et al., 2017). It is very necessary that we figure out the interaction between employee psychology, nudges, and external influences (Charness, 2004; Chen et al., 2024). Regrettably, academics have not paid as much attention to motivating employees to learn about green knowledge as it deserves, and there is an even greater lack of research that attempts to utilize nudges (either jointly or individually). This study fills the knowledge gap in this area based on the underlying logic of expectancy theory by linking employees’ psychological needs through the execution of a nudging experiment, which in turn stimulates proactive green knowledge learning. Our research posits that stimulating employees’ enthusiasm for green knowledge acquisition can effectively drive corporate green innovation (Bukoye et al., 2022). Green innovation, conceptually multidimensional, encompasses transformative activities across production processes, product development, managerial systems, and market strategies (Chiou et al., 2011). These organizational innovations demonstrate dual functionality: simultaneously reducing energy consumption and emissions while enhancing operational performance (Cuerva et al., 2014). As a systematic body of theoretical understanding and practical competencies pertaining to ecological conservation and sustainable development, the conceptualization of green knowledge has evolved from its initial environmental protection focus to a balanced paradigm emphasizing both ecological preservation and economic benefits (Gupta and Barua, 2018). Crucially, green innovation constitutes an outcome, whereas green knowledge learning serves as the instrumental means. The latter provides indispensable support for the realization of the former.

Papers exploring employees’ behaviors in green innovation reveal the vital precursors shaping their attitudes and actions. Individual factors such as green cognition and prospective earnings play a role (Chen et al., 2024). Organizational factors, such as the green innovation climate, resources, compensation, and appraisal system, as evidenced by Tulsi and Ji (2020), also sway employee involvement in green creativity and innovation. Additionally, the impact of social factors relating to environmental regulations and social oversight (Behera and Sethi, 2022) should not be overlooked. It is worth noting that many strategies aimed at fostering employees’ behaviors in green innovation are either costly or short-term, failing to fulfill the demands of sustainable development and resulting in a financial burden to some degree. Some scholars have conducted studies on how to encourage employees to engage in pro-environmental commercial activities at lower costs, based on the cognition of individual mental behaviors such as loss aversion (Dorschner and Musshoff, 2015) and peer pressure (Sayer and Cassman, 2013). As an economically feasible and psychologically receptive intervention, nudges are increasingly applied in various domains such as augmenting retirement savings, boosting education enrollment, and facilitating vaccination shots (Benartzi et al., 2017). The literature on nudges in production domains is less extensive than in consumption areas, particularly regarding promoting employees’ behaviors in green innovation. The ambiguity around the conceptual boundaries of green innovation and the complexity of staff behavior may be principal elements contributing to the limited research in this area. Existing literature suggests that nudge strategies are effective in promoting innovation among autoworkers (Tanaiutchawoot et al., 2019), foresters (Valatin et al., 2016), and project managers (Bukoye et al., 2022). Therefore, it is optimistic to assume that such strategies may also be applicable to promoting manufacturing employees’ green knowledge learning through similar individual psychological mechanisms. This study synthesizes theoretical results and practical explorations from previous studies to explore how different nudging tactics can be used to motivate employees to actively break out of their career comfort zones and learn green knowledge.

The distinctive feature of this study lies in its application of multiple behavioral nudging tools to measure shifts in employees’ behavioral tendencies toward green knowledge learning, using self-reported weekly time investment in green knowledge learning as the key metric. The study explored two nudge interventions: (I) providing information about injunctive social norms to stimulate peer comparison, and (II) releasing social status signals via compliments to encourage behavioral change. On this basis, the research also focus on evaluating the combined effects of both interventions and testing for the presence of crowding out effects. Against the backdrop of the important position occupied by nationalized business and the obvious power distance within firms, we divide the sample according to the dimensions of firm ownership and managerial position and conduct robustness tests and heterogeneity tests. The two tests help us to dig deeper into the differences in the effects of different nudging programs and may be one of the highlights of this study.

Starting with a literature review and hypothesis derivation, this study conducts a main effects analysis and heterogeneity analysis of survey data on employees’ green knowledge learning in Chinese manufacturing firms through a detailed research design and nudging experiment. Further, the study engages in an ethical discussion, emphasizing the breadth of application of boosting tools in economies under pressure from the dual goals of economic development and ecological protection. Finally, the study elucidates important application implications for business management practices and sustainable development, as well as contributions to academic research, and suggests limitations and future research directions.

## 2 Literature review and hypothesis development

### 2.1 China’s sustainable development pressures and efforts

**Figure 1:**
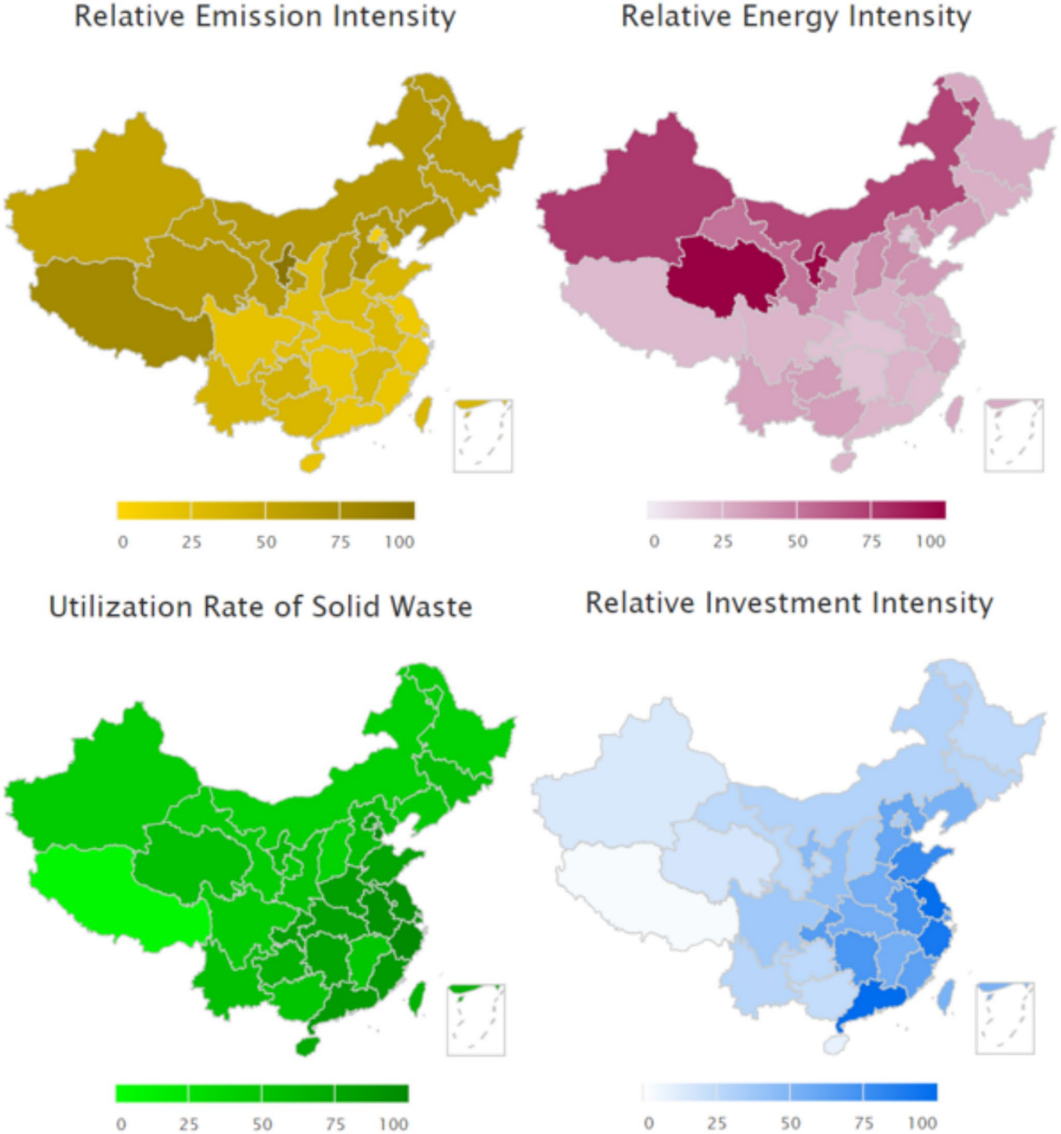
Environmental impacts of economic activities in China.

Academic Literature Shows Humanity is encountering severe ecological challenges in recent decades, including the greenhouse effect (Parmesan and Yohe, 2003), biodiversity reduction (Cardinale et al., 2012), and depletion of natural resources (Lotze et al., 2006). To achieve a sustainable balance between economic growth, social progress, and environmental protection, many governments have implemented green development initiatives (Rosen et al., 2008). Some companies also aim to execute environmentally friendly business practices, such as reducing emissions, enhancing resource efficiency, utilizing purification technology, and promoting circular production, in both advanced and emerging economies (Chiou et al., 2011; Cuerva et al., 2014; Gupta and Barua, 2018; Fernando et al., 2019). In addition, there are producers who are actively exploring key elements of sustainable manufacturing practices and sustainable development strategies (Koppiahraj et al., 2023). As a globally important manufacturing base, the indicators of energy consumption, emissions, waste recycling and investment in innovative activities in the production of Chinese enterprises show us how serious the ecological situation facing mankind is and how strong the determination to pursue green development is (see Figure 1). The upper two panels of Figure 1 illustrate the pressure levels of energy consumption and pollutant emissions across provincial-level administrative regions in 2022, with darker shading indicating higher pressure intensity. The lower two panels depict regional efforts in waste utilization and innovation investment, where darker coloration represents superior performance.^1^ This is like the ecological and employee health impacts of the mining industry in India (Marimuthu et al., 2023). There are many barriers to the implementation of green production in manufacturing in developing countries, as evidenced in Ponnambalam et al.’s (2023) study.

### 2.2 Nudging tools: social norms and social status

Regarding social norm nudging, some researchers propose utilizing peer comparison as a means of achieving diverse expected outcomes. The effectiveness of interventions has been demonstrated in practical applications, such as combating alcoholism (Werch et al., 2000), towel reuse (Goldstein et al., 2008), charitable donation (Shang and Croson, 2009), voting (Gerber and Rogers, 2009), contributions to online communities (Allcott, 2011), household energy saving (Allcott and Rogers, 2014), water conservation (Ferraro and Price, 2013; Jaime Torres and Carlsson, 2018), tax compliance (Hallsworth et al., 2017), and food consumption (Sparkman and Walton, 2017). Social norm intervention is commonly considered a cost-effective and beneficial approach to changing individual or group behavior by promoting conformity with prevailing social values, reducing the uncertainty inherent in decision-making, promoting social approval and respect, and enhancing psychological security (Farrow et al., 2017; Earnhart and Ferrart, 2021). Nonetheless, it is important to be wary of potential negative consequences, such as the backfire effect, which may arise from relying too heavily on peer comparisons or social norms. Beshears et al. (2015) discovered that informing individuals of their peers’ participation rate in a retirement savings example led to a corrosion of target object aspiration. There are several typical explanations for the emergence of negative effects, such as frustration induced by comparison, an insurmountable gap, dissimilar reference peers with the target object, forgetting information related to self-interest principle, and the presence of distractions (Dimant et al., 2020).

The implementation of social norms induces the transformation of individual motivation and behavior, which can be achieved through two typical ways, including informing respondents what other people do—descriptive norms or what other people think should be done—injunctive norms (Farrow et al., 2017; Dessart et al., 2019). Given that green innovation remains in its nascent stage globally, we chose to apply injunctive norms, which encourage rather than prohibit, to examine changes in employees’ green knowledge learning in industrial firms. Houdek (2024) provides useful lessons on how we can sensibly move forward with boosting and avoiding the associated pitfalls in organizational behavioral change. Specifically, this study shows why the injunctive norm of encouraging style can be more suitable for depicting individual green knowledge learning driven by the goals of carbon peaking and carbon neutrality. On the one hand, the conception and scope of green innovation is controversial as well as the action routine and critical process is multitudinous, so it may struggle to get the key point when informing the specific activities of their peers (i.e., leveraging descriptive norms) and may be in favor of recognizing important tasks and significant meaning when telling the expectation and values of cohorts (implementing injunctive norms) (Chen et al., 2010). On the other hand, as the backbone of industrial enterprises, occupational groups born in the range of 1970–1990s have stronger self-esteem and aversion to rough command, therefore, there is feasible for applying injunctive norms of encouraging genre rather than prohibiting type. Although the field of research on employee green knowledge learning is still in its infant stage, it may have unexplored differences from other individual behavioral traits. However, based on the successful application of social norms in facilitating individual behavior change, we anticipate that this nudging tool may also be useful in promoting employee green knowledge learning. We therefore propose:

*Hypothesis A:* The external modeling effect generated by social norms is transmitted to employees’ psychology, which is expected to enhance employees’ motivation for green knowledge learning.

The second nudge tactic of this study is social status through the attribution of compliments to industrial employees to examine the impact of the intervention on individual green knowledge learning. Employees’ pro-social actions can be stimulated by money or other material gifts, while they can also be encouraged by genuine recognition and compliments as part of the social system. In terms of pro-social behavior, some studies find that monetary rewards and material gifts help increase charity participation (Falk, 2007), reduce antibiotic use (Currie et al., 2013), and increase employees’ work enthusiasm (Akerlof, 1982; Fehr et al., 1993; Charness, 2004; Cohn et al., 2015). However, scholars who have researched the use of intangibles motivators, such as compliments and kindness, to stimulate good personal behavior are less relative to the above. Tidd and Lockard (1978) confirm that when employees in the service industry are friendly to customers, it helps them receive more tips. Subsequent studies provide evidence that complimenting service staff can achieve more qualitative and lasting effects on reciprocity than tipping (Kirchler and Palan, 2018; Lavoie et al., 2021). Julia et al. (2023) use status and reputation to examine their effects on positive reciprocal behavior among insurance brokers and suggest that attributing compliments to these professionals can improve their questionnaire response rate in the absence of clear commercial interest. Compliments can be found in everyday life and in the workplace to maintain a good relationship, which may cause confusion as to whether praise should be unhesitatingly classified as a nudge tactic.

According to the concept of social status signal, attributing compliments to individuals can release a kind of connotation that makes one feel a stronger competitive advantage compared to their peers. Moreover, the pursuit of higher social status is an instinct at the personal level, although it may not bring direct financial returns (Duesenberry, 1949), which comes from comparison with similar people (Bitektine, 2011). As a product of social relations (Patterson et al., 2014), status is highly integrated with hierarchical position and serves as a tool of social public evaluation. Manufacturing is an important part of global supply chains, where many outstanding talents are absorbed, and opportunities and challenges coexist. As such, if an employee occupies a higher status in the industry, it can increase the possibility of promotion post and increase wages, whether the industrial cycle is at a peak or a trough. In the context of the global wave of green innovation, superimposing the change of social evaluation system, we optimistically infer that manufacturing employees have a greater interest than in the past in pursuing an environmentally friendly reputation. In a word, if we give them commendation based on guiding future actions, these employees can be motivated to engage in green innovation, such as learning green knowledge, participating in green supply chain management, trying to solve new or old problems through creative production methods with environmental protection (Klewitz and Hansen, 2014). Against the backdrop of the global wave of sustainable development and increasing competitive pressures in the labor market, employees may be interested in green knowledge learning to meet corporate sustainability requirements and enhance their competitiveness in the industry. In other words, the industry may be able to enhance employees’ green knowledge learning if the industry puts a demand for green knowledge and grants a higher industry status to employees with green knowledge. Accordingly, we propose:

*Hypothesis B:* Social status enhancement is expected to enhance employees’ green knowledge learning by stimulating their desire to achieve.

### 2.3 Combination nudging and isolation nudging

Assuming again that the two boosting tools are effective, we investigated whether the size of treatment effect differs when managers simultaneously utilize two or more nudge tactics. Several influential studies have indicated that combination nudging could be effective in inducing pro-environmental behavior in a desired direction. Brandon et al. (2019) confirmed that implementing peak energy reports or home energy reports as intervention measures can decrease household electricity usage during peak load events and simultaneously executing both strategies does not result in a “crowd out” effect. Howley and Ocean (2022) found that informing farmers about the injunctive norm and social signaling helps increase their willingness to adopt new farming technology, specifically a smartphone app. Combining these two treatments results in a significantly greater impact. To assess the effects of isolation and combination based on the same psychological mechanism, this study employed four tactics to extend nudge analysis on individual behaviors. In this study we also validate the difference between joint and individual nudging through different boosting experimental designs. Specifically, we divided the combined group into two subgroups, “Norm + Status” group (first conducting peer comparison, then implementing compliments) and “Status + Norm” group (first conducting compliments, then implementing peer comparison). We argue that the size effect may be discrepant because of the different orders of stimuli, namely feeling the pressure first or feeling the pleasure first. In general, people seem to be more cooperative and positive when the mood is light, so we think it might be better to praise employees first (Bukoye et al., 2022). Because social norms and social status have different mechanisms of action on the individual’s psyche, their joint use may have complementary effects if properly operationalized. Further, in joint boosting, using the social status tool first for praise and then the social norm tool to provide comparative pressure may be more acceptable to subjects. We propose:

*Hypothesis C:* The selection of complementary tools for joint nudging may be more effective than individual boosting.

## 3 Materials and methods

### 3.1 Design

The aim of this study is to assess the effects of varied nudging interventions on employees’ green knowledge learning time. Our research strictly adheres to the ethical standards of behavioral science research. The experimental protocol underwent comprehensive evaluation by the Academic Committee of the School of Economics and Management at Lanzhou University of Technology, with particular emphasis on the following aspects: (I) psychological impact assessment of the intervention measures, (II) safeguarding mechanisms for voluntary participation, and (III) potential coercive effects arising from power dynamics in organizational settings. The informed consent procedure followed a two-phase implementation framework: Prior to the experiment, participants were fully informed via an independent online platform about the research objectives, experimental procedures (including randomization mechanisms), data usage scope, and privacy protection measures. It was explicitly stated that participation was voluntary and anonymous, and that involvement (or lack thereof) would not affect performance evaluations. All procedures complied with the Ethical Principles of Psychologists and Code of Conduct (APA) and the International Labour Organization (ILO) ethical standards for workplace research. Periodic ethical reviews were conducted throughout the study to ensure full compliance during the entire experimental process.

**Figure 2:**
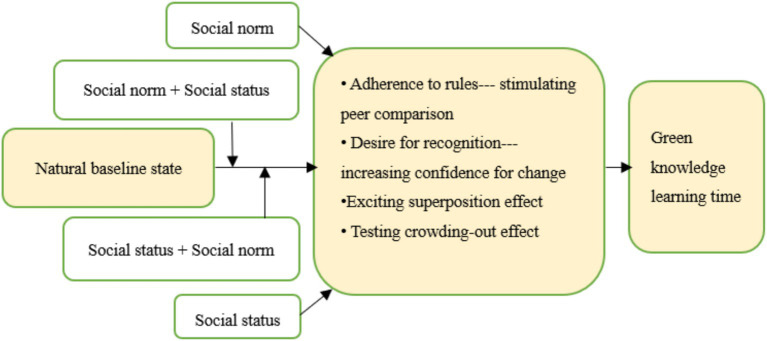
Experimental design logic.

**Figure 3:**
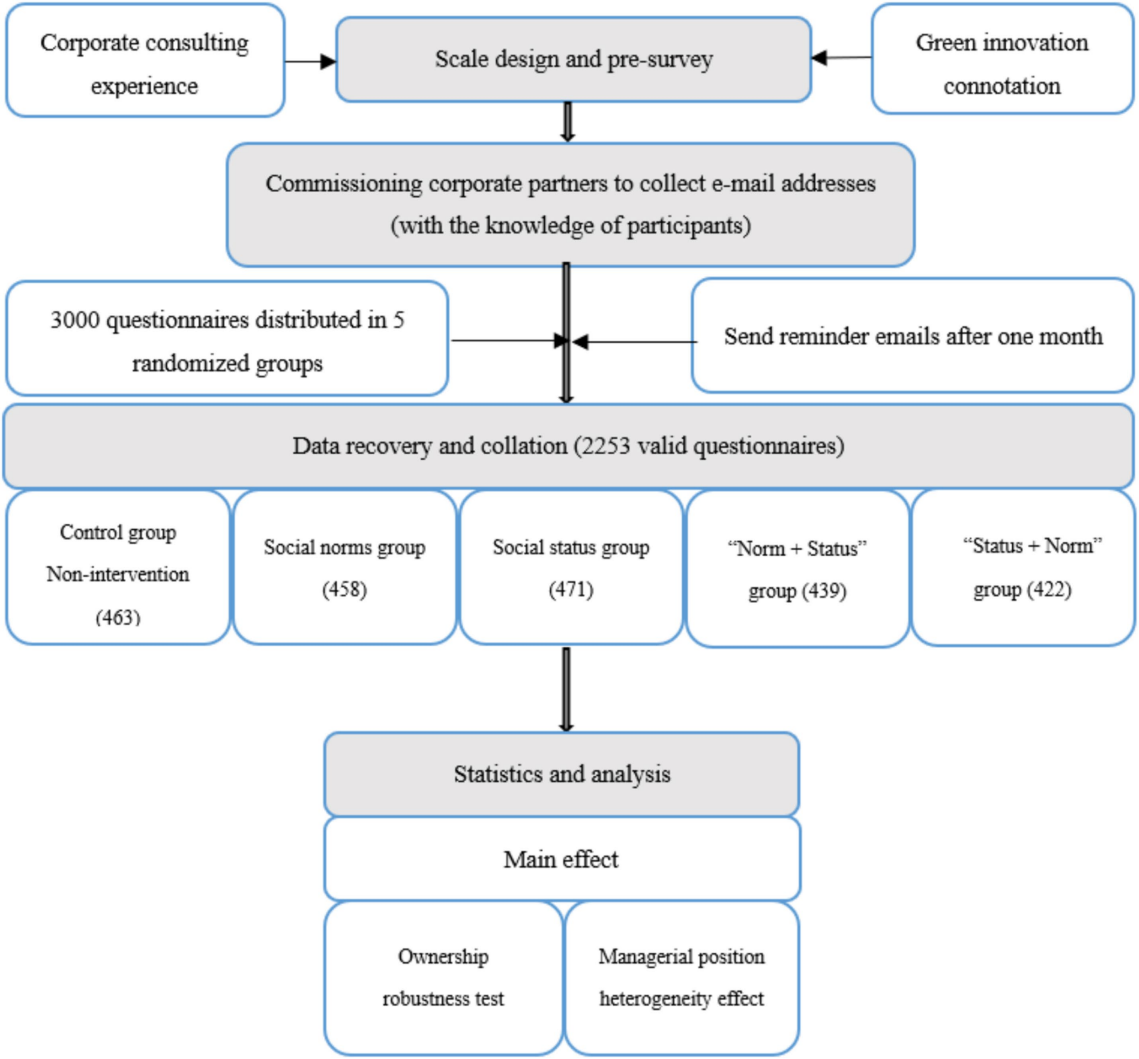
The overall research process.

The subjects were randomly divided into five groups: control group, social norm group, social status group, “Norm + Status” group, and “Status + Norm” group (see Figures 2, 3). The random grouping of the boosted subjects is mainly based on the following considerations. The first is to eliminate the influence of heterogeneous factors, such as age, gender, years of working experience, nature of the working enterprise, geographical environment, etc., which are likely to interfere with the experimental results when the intensity of the above factors is large. Second, the number of employees included in each group is larger and the characteristics of all employees are more diversified, which enables us to test the effectiveness of the booster tool in a more open environment. Third, during the analysis of our results, we selected the nature of business ownership and the management level of the employees for targeted validation, which balances general and specific analytical needs.

In the control group, we emphasized the environmental threat of human social development and the necessity of promoting green innovation, as well as indicated that studying green knowledge is the entry point of personal participation in green innovation. The respondents rated the preferred extra time to learn green knowledge per week on a scale from 0 to 10. We temporarily set the maximum time as 10 h per week, due to the high work intensity of employees in manufacturing companies, and to protect the initiative for learning green knowledge among the surveyed employees. Reassuringly, our goal was to examine the relative effects of diversiform nudge tactics rather than to measure study time accurately, so setting the 0–10 scale did not conflict with the goals of this research. In the social norm group, we added a paragraph at the beginning of the questionnaire text to demonstrate the practice attitude of peers, in which we had given a favorable rating of 95% based on our experience in providing consulting services to manufacturing companies. In the social status group, we added a paragraph to praise the respondent’s action and painted a bright career future near the end of the text. In the “Norm + Status” group, we first informed the respondents about the actions and attitudes of their peers in the opening section and then praised and encouraged them at the end. In “Status + Norm” group, however, we began with praise and then applied pressure. The complete questionnaire can be found in Appendix.

The core problem with our design was how much extra time per week employees would be willing to spend studying green knowledge. We wanted to test three different types of effects: (I) in the control condition, what was the effect size of implementing the social norm and social status interventions separately, (II) in contrast to the independent stimulus, whether there would be a crowding out effect of implementing the combined intervention, and (III) in the joint interference, what would happen because of the different order of implementation of social norm and social status. If there was a crowding out effect, then the combined effect would be less than the sum of the effect of implementing social norm and social status, respectively. The design of our boosting experiment is also in line with the design of Brandon et al.’s (2019) energy use boosting experiment and Howley and Ocean’s (2022) innovation application nudging experiment, whose explorations in the areas of joint and individual nudging provide useful lessons for this study.

### 3.2 Procedure and participants

For the sake of clarity, after designing the questionnaire, we randomly conducted two rounds of pre-surveys among students as well as our relatives and friends, including different ages, genders, and educational levels (see Figure 3). Through continuous perfection, we believed that the questionnaire statements could clearly express our true intention. In addition, we obtained information other than commitment learning time, such as the ownership of the company (state-owned or private) and the employee’s position (manager or non-manager). To reduce the respondents’ burden and avoid negative irritation that might lead to biased responses, we did not ask respondents about their age, gender, education, location, etc. Meanwhile, we argued that it would increase the difficulty and cost of the nudge intervention to take too much account of demographic characteristics. Frankly speaking, it is impossible to develop a variety of nudges for the same occupational group, and it is extremely costly if someone persists, which may also violate the principle of nudging.

Prior to contacting interviewees, we conservatively calculated the minimum sample size to meet the research requirements according to G*Power 3 and 3.1 (Faul et al., 2007, 2009). In general, the effect size related to behavioral intervention research is small (Cohen’s *d* < 0.2, or 0.2 < *d* < 0.5), we used *d* = 0.2 as well as 5 percent level (two-sided) and 0.8 power level as the calculation basis. For each group of our study, we needed at least 394 samples, or a total of 1,970 valid questionnaires. To ensure an adequate sample size, we intended to administer 3,000 questionnaires, a process that required logistical support from the university’s alumni network. We selected 851 manufacturing firms from the alumni enterprise database, assigned each a unique identifier, and randomly chose 300 for our study. Two of the paper’s authors conducted individual phone calls with the alumni representatives of these 300 firms, explaining the purpose of the experiment and requesting each alumnus to provide 10 employee email addresses from their respective companies. The email addresses collected were then grouped based on the alphabetical order of their initials, with each group allocating 600 questionnaires. Employees from the same firm were randomly distributed across different groups to ensure randomization. This approach also helped mitigate potential interference from participants’ demographic characteristics on the experimental outcomes. The survey was conducted between October and December 2022, during which we sent a questionnaire along with a thank-you letter to each email address. One month after the survey invitation, we sent another email to remind the respondents. To ensure respondents’ informational privacy, all survey responses were directly submitted to the corresponding author without intermediary transmission (e.g., via email collectors). Consequently, no individuals other than the research team had access to completed questionnaires, while respondents’ identities remained anonymized throughout the study. Furthermore, this research strictly adhered to ethical guidelines by: (I) maintaining full transparency in all research communications, (II) avoiding categorical labeling of participants, and (III) preserving complete behavioral autonomy. In the end, we took back 2,253 questionnaires (75.10% effective response rate), which included 463 questionnaires of control group (77.17% effective response rate), 458 questionnaires of social norm group (76.33% effective response rate), 471 questionnaires of social status group (78.50% effective response rate), 439 questionnaires of “Norm + Status” group (73.17% effective response rate), and 422 questionnaires of “Status + Norm” group (70.33% effective response rate), respectively. State-owned enterprises respondents are 1,051, 46.65 percent proportion, and managers respondents are 1,119, 49.67 percent proportion.

## 4 The results

### 4.1 Main effects

We analyzed the overall survey samples and compared them with the effects of five experimental groups that were similar in sample size. Table 1 shows detailed statistical results. In the control group, without any intervention, the mean value of preferred extra time was 4.432 in the range of 0–10 h. In the social norm group, study time increased to some extent, with a mean of 4.795. We found an increase of 8.12 percent according to the sample t-test and an effect size of 0.136, more strikingly, it was statistically significant (*t* = 2.904, two-sided *p* = 0.004). In the social status group, the results showed a higher mean (4.909), a more pronounced increase (10.72 percent), a larger effect (0.163), and a stronger level of significance (*t* = 3.498, two-sided *p* < 0.001). An exciting consequence was that nudging strategies can achieve a better intervention effect than the baseline level, even when used alone. Hypothesis A and hypothesis B are confirmed. This fits with Sanchayan et al.’s (2023) conclusions in their study of online boosting of sustainable behavior in individuals. This strongly suggests that, as in other fields, the implementation of nudging tactics can significantly improve employees’ green knowledge learning. It also gives us more confidence to further study the combined effect, and that is, whether there is a crowding out effect or not, we have something to gain.

**Table 1:** Core statistical indicators for main effects analysis.

Statistic	Control	Norm	Status	Norm + Status	Status + Norm
Sample size (N)	463	458	471	439	422
Mean value	4.432	4.795	4.909	5.355	5.545
Standard deviation	1.876	1.946	2.131	1.851	1.817
Cohen’s *d* (Effect size)		0.136	0.163	0.341	0.421
Treatment mean vs. Control mean		0.360	0.475	0.907	1.104
*p* value vs. Control (two-sided)		0.004	<0.001	<0.001	<0.001

In addition, we conducted the analysis of combined treatments from two aspects of “Norm + Status” group and “Status + Norm” group. In “Norm + Status” group, there was a stunning investment of average additional learning time of 5.355 h, as well as a dramatic reinforcement of 20.46 percent. T-test showed that Cohen’s *d* is 0.341, which exceeds the level of 0.2 and is highly significant (*t* = 7.136, two-sided *p* < 0.001). In the “Status + Norm” group, there was a result of both joy and fear for us. It was natural to be happy because the statistics were so good (mean = 5.545, 24.91 percent increase, Cohen’s *d* = 0.421, *t* = 8.641, two-sided *p* < 0.001). Hypothesis C is confirmed.

**Figure 4:**
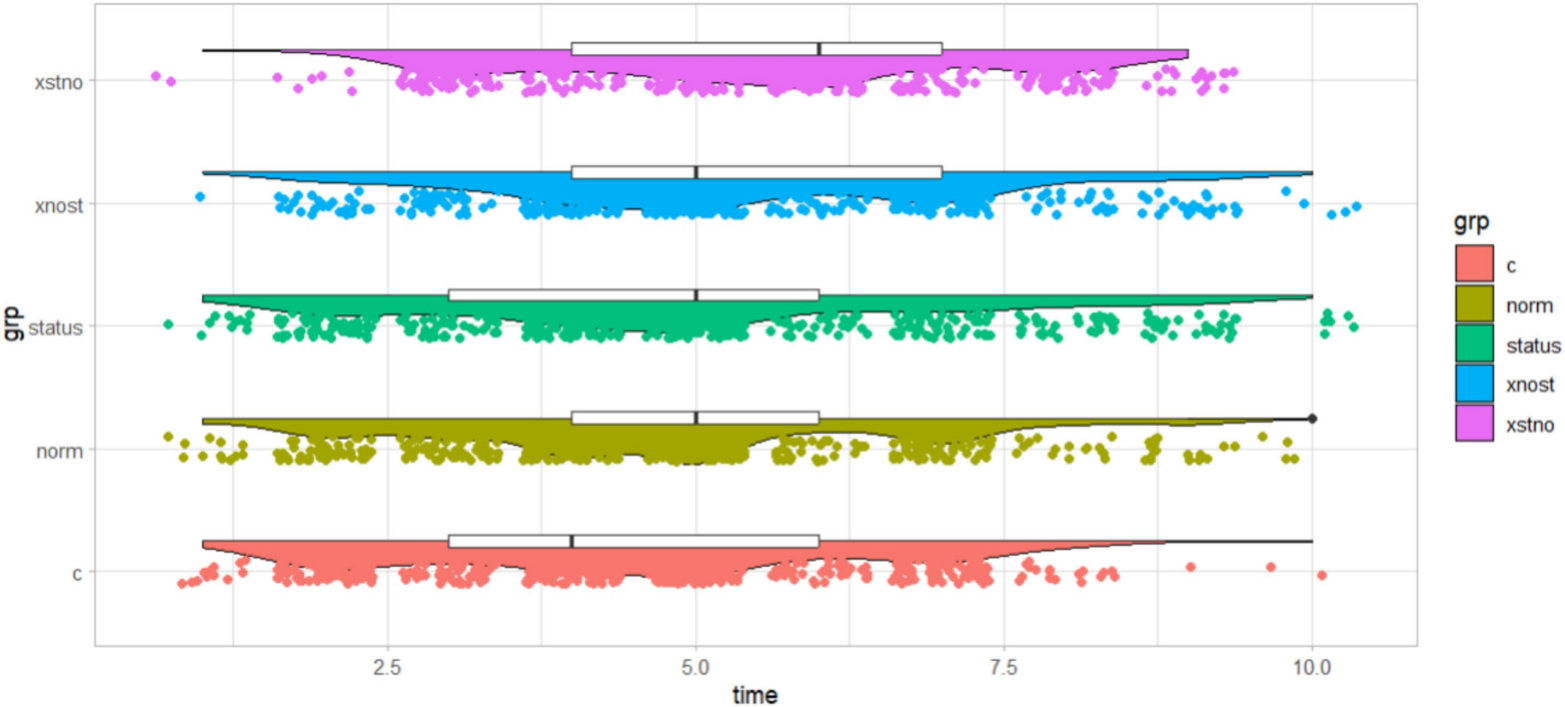
Effects of different nudging strategies.

It may be puzzling when it comes to fear after the event. The truth is clear that if we do not carefully divide the combined group into “Norm + Status” group and “Status + Norm” group, some valuable conclusions may be obscured. Compared with the former, the latter showed a more outstanding nudging effect in stimulating the green knowledge learning of manufacturing employees. It seems that the type of reward before pressure, i.e., social status intervention first and then social norm intervention, can help to achieve a more significant effect in the context of green innovation. Overall, we sorted these nudging tools in order of mean value from small to large in our study scenario: social norm (denoted by “norm”), social status (denoted by “status”), “Norm + Status” (denoted by “xnost”) and “Status + Norm” (denoted by “xstno”) in Figure 4. It is believed that nudging strategies are effective and low-cost instruments under the circumstances of promoting green knowledge learning of manufacturing enterprises, as well as a strong complement to fiscal, monetary, and industrial policies of governments and financial institutions, when they are used either alone or in combination with each other. In addition, the combined effect of using two nudge strategies simultaneously is greater than the individual effect of implementing social norm or social status separately, while it is greater than the sum of both independent effects. Therefore, we found no crowding out effect in both combined groups, which also means that we can further explore the relationship between multiple nudging tactics and selectively apply them in green knowledge learning.

### 4.2 Robustness tests

To verify the robustness of the nudging experiment, we peel the 2,253 respondents based on the ownership of the enterprise and repeat a series of analyses as above. More critically, this stripping provided us with a channel to thoroughly understand the differences in the effect size as well as other key indicators between state-owned enterprises and private enterprises. This robustness test, unlike others such as gender, age, location, and educational attainment, more fully reflected the status quo of China’s manufacturing industry, in which state-owned enterprises occupy a significant proportion and play an important role (Zhang et al., 2024). In mainland China, data from the National Bureau of Statistics (NBS) show that private companies are far more enthusiastic about innovation activities than state-owned enterprises (SOEs). In 2022, 20.6% of SOEs realize product innovation, compared to 35.5% of private enterprises. The share of state-owned enterprises realizing process innovations is 30.3%, compared to 40.8% for private enterprises (see text footnote 1). The ratio of state-owned enterprises and private enterprises were almost identical in this study, the former was 53.35% and the latter was 46.65%. Table 2 shows the main indices of the robustness test.

**Table 2:** Robustness tests based on business ownership.

Statistic	Control	Norm	Status	“Norm + Status”	“Status + Norm”
Sample size (N)	259/204	245/213	238/233	232/207	228/194
Mean value	4.479/4.373	4.739/4.859	4.824/4.996	5.263/5.459	5.483/5.619
Standard deviation	1.868/1.888	1.922/1.976	2.145/2.118	1.772/1.935	1.828/1.806
Cohen’s *d* (Effect size)		0.081/0.161	0.105/0.188	0.296/0.403	0.377/0.490
Treatment mean vs. Control mean		0.216/0.456	0.303/0.534	0.763/1.098	0.982/1.268
*p* value vs. Control (two-sided)		0.205/0.022	0.107/0.008	<0.001/<0.001	<0.001/<0.001

The data before “/” are affiliated with state-owned enterprises and after “/” are indicators of private businesses.

**Figure 5:**
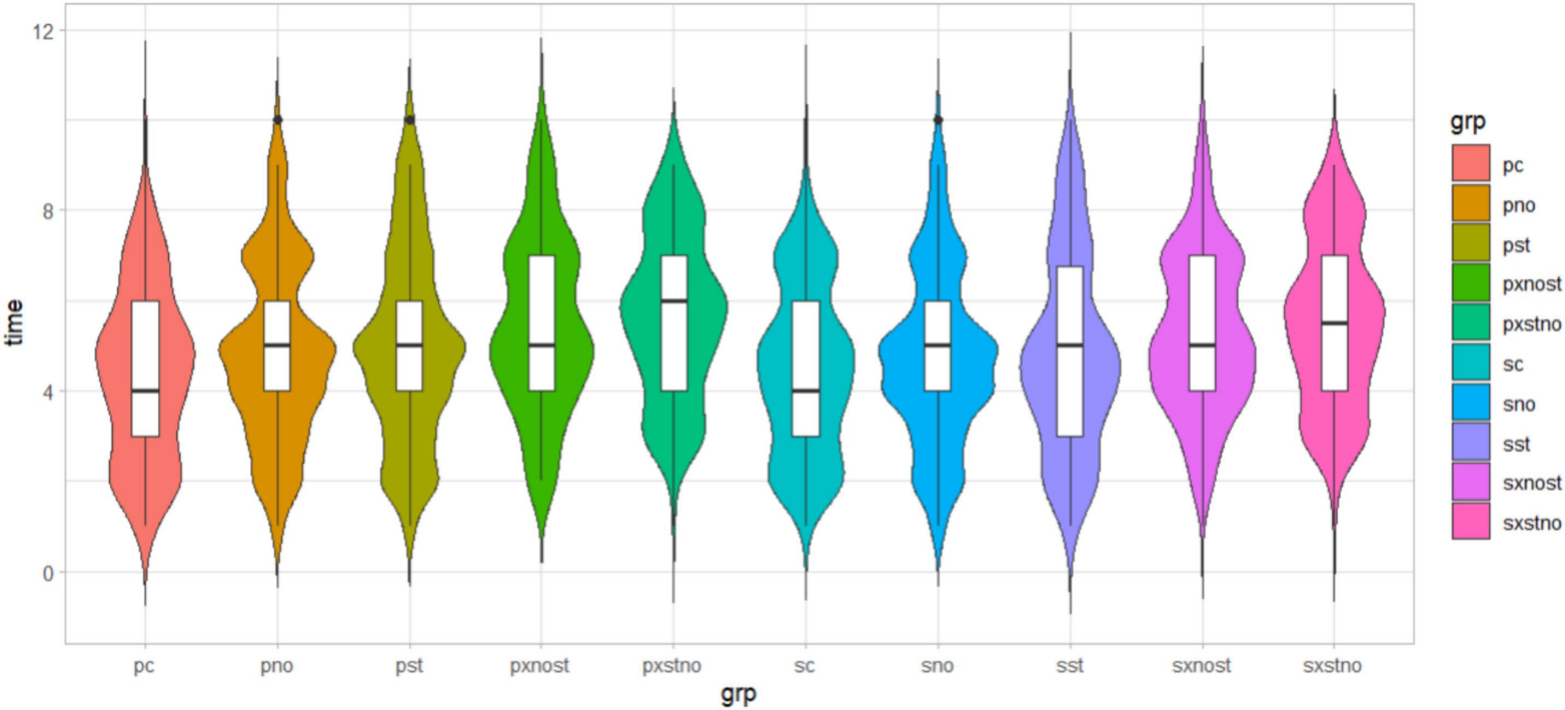
Nudging effects of different ownership.

Among the 1,202 state-owned enterprise respondents, we found roughly the same mean and standard deviation as the full sample, and some difference in effect size and *p*-value. Meanwhile, there were also some potentially revealing gaps (see Figure 5) between state-owned enterprises (control group, social norm group, social status group, “Norm + Status” group, and “Status + Norm” group are denoted by “sc,” “sno,” “sst,” “sxnost,” and “sxstno,” respectively) and private firms (denoted by “pc,” “pno,” “pst,” “pxnost,” and “pxstno,” respectively).

On the one hand, the mean value of private firms revealed a greater intervention effect than the other two samples, either alone or in combination, the effect size is also larger. On the other hand, the *p*-value of private enterprises showed better significance than state-owned enterprises. It is a pity that the latter did not pass the significance test on norm treatment and status treatment at the level of two-sided 0.05 in the sample of SOE employees. This could mean that it is more adaptable to promote behavioral change among employees of private enterprises using nudge strategies. These findings reaffirm that the simultaneous use of social norm and social status does not induce a crowding out effect in nudging behavior change, and the effect of combination is more effective than separation. In the group “Status + Norm,” the effect values for state-owned enterprises and private enterprises reached 0.377 and 0.490, respectively.

### 4.3 Heterogeneity effects

Given the behavioral differences between managers and non-managers (Nyberg et al., 2015), we conducted heterogeneity analysis to explore whether such discrepancy exists in the green innovation scene. We expressed this confusion in the introduction section of this paper, based on the motivational orientation of personnel at different levels in the business organization. Another reason that encouraged this study to conduct heterogeneity tests is our confidence in sample size. We divided the total sample into manager subgroup and non-manager subgroup, in which respondents of both teams were almost equal (49.67 percent versus 50.33 percent). In one control group and four treatment groups, respondents differ by a maximum of 4.78 percentage points and a minimum of 1.75 percentiles. See Table 3 for details.

**Table 3:** Heterogeneity analysis—staff position.

Statistic	Control	Norm	Status	“Norm + Status”	“Status + Norm”
Sample size (N)	237/226	225/233	229/242	209/230	219/203
Mean value	4.300/4.571	4.898/4.695	4.856/4.959	5.450/5.270	5.639/5.443
Standard deviation	1.855/1.892	1.981/1.911	2.088/2.175	1.837/1.864	1.865/1.763
Cohen’s *d* (Effect size)		0.213/0.052	0.198/0.131	0.460/0.283	0.492/0.304
Treatment mean vs. Control mean		0.573/0.142	0.572/0.376	1.129/0.712	1.320/0.872
*p* value vs. Control (two-sided)		0.002/0.432	0.003/0.050	<0.001/<0.001	<0.001/<0.001

The data before “/” are affiliated with managers and after “/” are indicators of non-managers.

**Figure 6:**
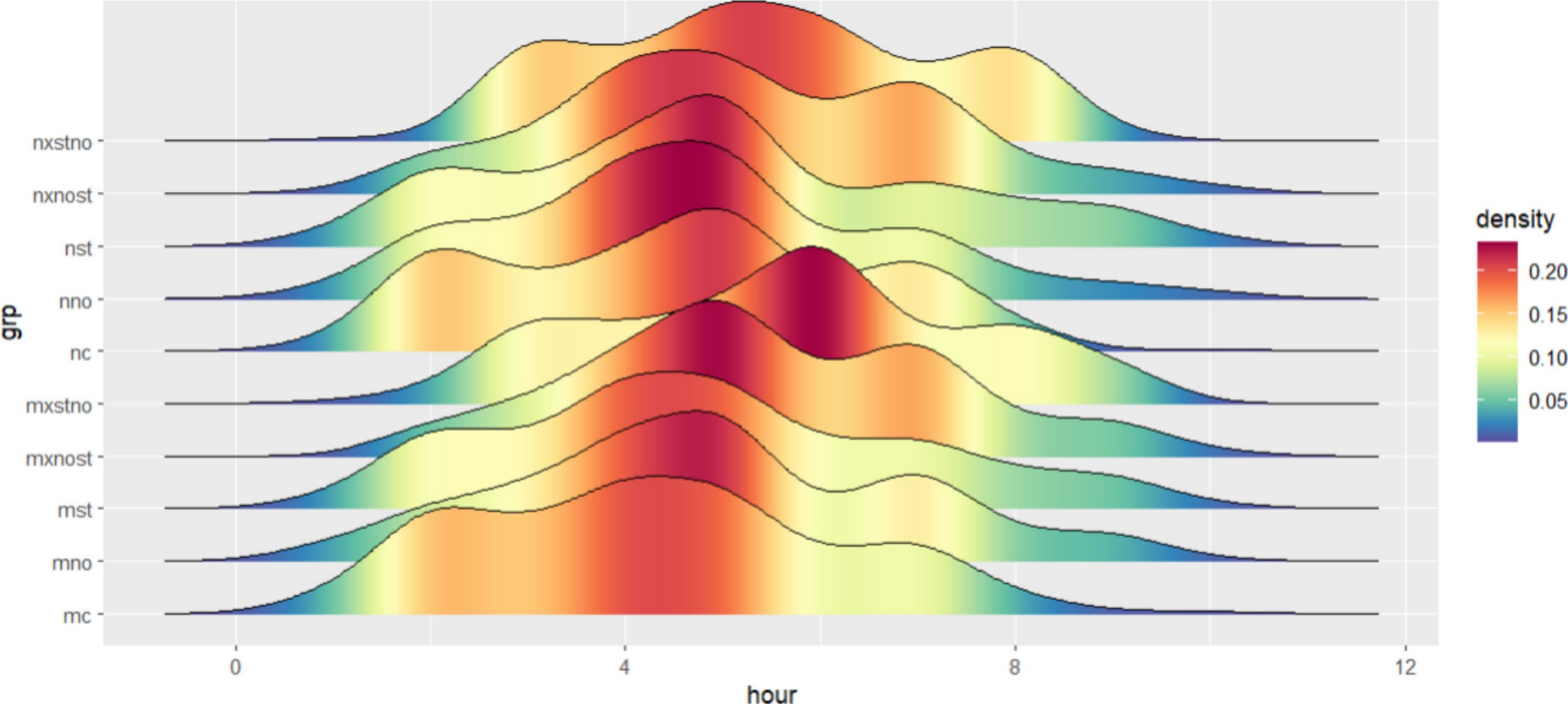
Nudging effects of managerial and non-managerial groups.

On average, the initial level of managers is lower than that of non-managers. However, managers’ level was increased more significantly by social norm stimuli, combined treatment of “Norm + Status” and “Status + Norm,” not including the social status group. Figure 6 shows the effectiveness of the different boosting schemes in a peak-to-peak diagram, where the red peaks represent the areas with the highest concentration of data.

The control group, social norm group, social status group, “Norm + Status” group, and “Status + Norm” group are denoted by “nc,” “nno,” “nst,” “nxnost,” “nxstno” in the community of non-managers, and denoted by “mc,” “mno,” “mst,” “mxnost,” “mxstno” in the community of managers. On Cohen’s *d*, managers’ scores were higher than non-managers in four experimental groups, with the gap widening from 0.067 (social status group) to 0.188 (“Status + Norm” group). On *p*-value, managers in each group all passed significant test at two-sided 0.05 level, non-managers did just as well in “Norm + Status” group and “Status + Norm” group. So far, comparing the above three aspects, we argued that the effect was better in managers than non-managers implementing nudging tactics.

### 4.4 Discussions of the experimental process

To avoid counteracting each other, we still need to pay attention to a few details in accordance with the operational procedure of the experimental study. First, the stimulus sequence is a crucial factor that can be easily overlooked. We all know that effective communication must be done in a relaxed atmosphere, or at least not in a way that makes the other person resistant. Compliments before pressure, we strongly believe, create a more conducive environment for collaboration than the opposite approach, especially when communicating with strangers online. The empirical results support our view, and this experimental design is also a value of this paper. Second, sentence expression is a key factor in ensuring the smooth conduct of research due to the differences in language habits in diversified cultural backgrounds. Experimental designers might do well to make subjects feel concise, clear, sincere, and friendly when implementing the nudging strategy, especially when we make compliments. Giving respondents a sense of both responsibility and accomplishment in behavioral tests is a challenge for any research team (Allcott and Mullainathan, 2010). Third, the number of items plays an imperceptible role. Frankly, our team members are often the subjects of various studies and especially hate surveys with too many questions. Although we do not express this dislike verbally, our actions are honest, i.e., we answer items mechanically without thinking and strive to complete the questionnaire in the shortest time possible. Based on empathy, we recommend designing as few central questions as possible and not making the options too complex. For example, this study did not include demographic characteristics such as age, education, and gender because it is impossible to break down the implementation of the nudging strategy into such detail from a cost and operational perspective. Finally, partnering with reputable platforms or channels is an important guarantee that they will enroll active participants in the experiment, which helps reduce research costs and improve the effective questionnaire rate. Although partners may pool highly cooperative employees based on high efficiency, it does not undermine the credibility of the results because the conditions are the same for all experimental groups.

### 4.5 Discussion of ethicality

Our nudge of green knowledge learning for corporate employees is primarily based on employee perceptions, such as observations of the Earth’s greenhouse effect, concerns about the depletion of non-renewable energy sources, and perceptions of pollutant emissions from production. Based on cognition, employees’ needs for socialization, respect, and achievement are utilized to influence their choice architecture by encouraging the application of type social norms, using the provision of information rather than coercive commands. The nudging experiment consists of the mainstream ethical and moral norms of human society and strengthens employees’ choice autonomy to a certain extent. First, in the competitive and motivated corporate workplace environment, especially in industries where productivity enhancement is the action guideline, the corporate management style very often reflects command or obedient characteristics, and it is difficult for employees to have the opportunity to rationally question management decisions. The application of boosting tools retains employees’ free choice to learn green knowledge and there is no punishment for employees who do not actively participate in green innovation. Driving employees to break out of their comfort zones mainly caters to their values of pursuing excellence and social responsibility. Second, a significant portion of our experimental sample is managers. There is a high chance that our boosting experiment will inspire managers. Managers may reflect on what kind of leadership style is both effective and acceptable to their employees (Tonglet et al., 2004). Boosters that appeal to the glowing human values of employees have a chance to become a powerful weapon in the managerial toolbox. Optimistically, more and more managers will consider adopting facilitation as the experimental use of facilitation in business management continues. Our study indirectly enhances employees’ self-efficacy, career fulfillment or job satisfaction, and psychological well-being through the medium of management practices. Finally, through subtle boosting of employees’ willingness to learn green knowledge, employees will have a stronger understanding of green innovation. Instead of setting the scope of green innovation, we encourage employees to rethink their workflow or work content through the lens of green innovation in their own jobs and encourage them to explore on their own ways of working that are both ecologically and economically efficient. This approach not only reserves green innovation momentum for society but also enhances employees’ sense of value and production ethics, i.e., realizing a balance between economic and ecological benefits.

## 5 Conclusions and discussions

### 5.1 Conclusion

In this research, we investigated the effect of diversiform nudging modes on motivating employees’ green knowledge learning through randomized experiments among five groups. Not only were individual effects evaluated, but two types of combined effects were also examined. There is a striking increase when the social norm or social status interventions are implemented separately. Our study builds upon Farrow et al.’s (2017) and Dessart et al.’s (2019) conceptual definitions of descriptive norms and injunctive norms, extending the latter’s research to the context of green innovation. Moreover, the studies by Lavoie et al. (2021) on service personnel and Julia et al. (2023) on insurance brokers regarding behavior changes induced by praise nudge provide valuable references for our research on green knowledge learning among manufacturing employees and bolster confidence in extending nudging tools such as praise and social status to other behavioral domains within green innovation contexts. The effect size of the social norm is 0.136 and that of the social status is 0.163, which is an increase of 8.12 percent and 10.72 percent, respectively, over the control level. Meanwhile, both types of nudges are statistically significant, with two-tailed *p*-values less than 0.01. Like Jennifer et al.’s (2024) study, both reflect the important role of person-centeredness in fueling experimentation. In two other combined effects experiments, we found a more pronounced nudging role, which is the effect size increment of 20.46 percent or 24.91 percent compared to the baseline state, and both *p*-values less than 0.01. We verified two important findings that there is no crowding out effect and praise before pressure is more effective. The findings of this study align with and extend the seminal work of Brandon et al. (2019) on energy use boosting experiment and Howley and Ocean’s (2022) research on innovation application nudging experiment, collectively contributing to a coherent theoretical framework.

After the main effect analysis, we conducted robustness test and heterogeneity test for subdivided samples of firm ownership and employee position. These two methods of sample division are useful for studying green knowledge learning, based on the reality that state-owned manufacturing enterprises account for a certain proportion and management positions influence employees’ attitudes and behaviors. Our results also show significant differences in the sub-sample analysis under the premise of supporting no crowding out effect. Robustness test echoes Liu et al.’s (2024) view of promoting responsible corporate environmental engagement in China. Of course, this result may not be convincing due to the limited sample size and can be further confirmed by increasing the sample size and elaborating the experimental design. The results of heterogeneity test could be an indication to practitioners that motivating managers with nudges may be more valuable. This echoes Ren et al.’s (2024) research on firms’ green innovation behavioral boosts. It is a little unfortunate that this study did not distinguish the sample of senior managers, middle managers, and junior managers, which could also be one of the directions of our follow-up research. Moreover, it is possible to implement the nudging strategy according to the type of firm and employee position from the perspective of cost saving or ease of operation. From a micro-level analytical perspective, this study contributes a cost-effective and flexible managerial framework for motivating employee-driven green knowledge learning while theoretically extending four established motivational paradigms—Expectancy Theory (Vahe and Shen, 2024), Achievement Motivation Theory (Rachmatullah et al., 2021), Maslow’s Hierarchy of Needs (Carpenito-Moyet, 2003), and Herzberg’s Two-Factor Theory (Alrawahi et al., 2020)—into the organizational sustainability domain. Macro-level implications suggest this research holds substantial transformative potential for reconciling corporate profit-ecology tensions and advancing the United Nations Sustainable Development Goals (Behera and Sethi, 2022; You et al., 2015).

### 5.2 Theoretical implications

The indeterminate interaction effects arising from the concurrent application of multiple nudges on individual psychological cognition processes underscore the imperative for our investigation into green knowledge learning. To improve treatment effectiveness and reduce costs, we focused on analyzing the combined effect of implementing injunctive social norms and social status signals. First, the use of social norms and social status in this study echoes the ideas proposed by the hierarchy of needs theory (Carpenito-Moyet, 2003), in which social norms are closely linked to employees’ social needs and social status is linked to employees’ respect needs. Further, employees’ behaviors of learning about green knowledge and breaking out of their occupational comfort zones through self-directed learning are also aligned with the self-actualization needs proposed by the hierarchy of needs theory. The use of the combination of social norms and social status satisfies the three needs of employees to receive incentives, which also provides a theoretical explanation and rationale for the superiority of the combination over isolated use. Secondly, in relation to the two-factor theory (Alrawahi et al., 2020), the two enablers mainly echo motivational factors rather than health care factors. The use of the combination of social norms and social status is better able to satisfy the motivational factors of achievement, appreciation, challenging work, increased job opportunities, and opportunities for growth and development. Further, how to match more efficient and cost-effective health care factors, corresponding to boosting tools that aim to provide motivational factors, also suggests new topics for theoretical reflection. Third, echoing achievement motivation theory (Rachmatullah et al., 2021), social norms are directly related to the need for conformity, and social status is directly related to the need for achievement, and the combination of the two enablers can satisfy the needs of employees in more diversified dimensions and show a more powerful driving effect than the use of the two enablers in isolation.

### 5.3 Practical implications

Building upon the empirically established efficacy of individual nudging strategies, we posit that stakeholders—encompassing policymakers, scholars, and corporate leaders—should prioritize investigating the synergistic effects of nudge combinations. A systematic differentiation of nudge attributes is methodologically essential, given the demonstrable heterogeneity in their underlying psychological activation mechanisms (Lades and Delaney, 2020). In this research, we compared the effect of psychological interventions on respondents by implementing the combination of social norm and social status, whether it is pressure or reward first. In other words, although we implemented various experimental stimuli, the target point was accordant, that is, to motivate the social comparative psychology of employees (Gul and Ak, 2018). The respondents viscerally felt that if they did not conform to the behavior of other majority members, especially their peers, they might face obstacles from the mainstream group, and this is the common nature of humans and similar social animals. This behavioral trajectory exhibits congruence with the empirical findings of Brandon et al. (2019) and the theoretical framework advanced by Howley and Ocean (2022). Determining the existence of such ostensibly enhanced effects when employing divergent psychological mechanisms for intervention presents notable methodological challenges. In the scenario of stimulating employees’ green knowledge learning, if we simultaneously trigger productivity and innovation, or financial performance evaluation and ecological benefits, there is likely to be a crowding out effect. Once extrusion occurs, it can reduce the push effect in the short run and make the subject immune to the nudge in the long run.

The empirical findings of this study yield substantive implications for global industrial advancement and green innovation across diverse geographical contexts. First, although this paper mainly focuses on the employees of Chinese manufacturing companies as the experimental sample, the boosting tools used closely echo the mainstream employee motivation theories such as the Hierarchy of Needs Theory, the Two-Factor Theory, and the Need for Achievement Theory. These well-established motivation theories emerged through systematic observation, synthesis, and empirical validation across diverse temporal, geographical, and industrial contexts, thereby substantiating the potential for broad applicability of the motivational instruments examined in this study across multiple regions and industrial sectors (Della and Linos, 2020). Second, our research process is also consistent with the boosting process in different regional samples over time, and the standardization of the boosting scheme provides a solid foundation for the generalization of the findings of this study. Third, numerous developing nations, particularly China, confront the fundamental dilemma of reconciling economic growth imperatives with ecological conservation requirements (Simon and Eric, 2007). In the face of the arduous historical mission of green innovation, it is difficult for many regions to provide sufficient incentives to enterprises in terms of policies, funds, and taxes, which provides a wide space for the use of low-cost boosting tools. Finally, although there are differences in the history, culture and industrial characteristics of countries around the world, at the macro level, human beings share the same desire to win social recognition and respect, to pursue better personal development, and to look forward to the improvement of the ecological environment. This investigation substantiates and extends the conceptual framework and empirical findings of Kalamaras et al. (2024) regarding boosting interventions for residential energy efficiency in developed economies, thereby demonstrating the extensive applicability spectrum of boosting methodologies across diverse contexts. Our promotion program is well suited to the underlying needs of human survival and can therefore be used as a universal reference across cultural, regional and industrial differences.

### 5.4 Limitations and future scopes

We explicitly acknowledge the inherent methodological limitations that characterize all empirical research endeavors. In designing and executing the booster experiment, we faced several obstacles and puzzles, which pointed the way to continued research in the future. First, we used the method of sending text via e-mail for the nudging intervention, and although we made fine refinements to the text semantics and reminder e-mails, it is inevitable that there may still be cases in which respondents are unclear about the semantics or negatively cooperate with the experiment. In the future, we will try to use field experiments to enhance the control of the experimental sessions, which may lead to more credible conclusions. Second, notwithstanding our deliberate exclusion of demographic variables (e.g., age, educational attainment, income, and geographical location) to minimize respondent burden and enhance participation rates, this methodological choice may have compromised our ability to detect potentially significant patterns. Specifically, climate-conscious younger cohorts—socialized in an era of heightened environmental discourse—likely exhibit greater cognitive engagement with sustainability issues, while highly-educated individuals may demonstrate stronger orientation toward economy-society-ecology synergies. These observed limitations underscore the imperative for future research to systematically incorporate sociodemographic analyses when examining employee behavioral responses within green innovation paradigms. Third, while the current study employs a relatively robust sample size, constraints in data structuring and analytical sophistication may limit comprehensive data mining capabilities, potentially obscuring additional empirically significant relationships. In the future, we will continue to improve the level of data analysis and try to use more diversified statistical analysis methods to mine the underlying logic of the data. Finally, while social norms and social status demonstrate efficacy in enhancing employees’ green knowledge learning engagement, our study may not have captured potentially more impactful behavioral enablers. Future research should systematically explore a broader spectrum of behavioral interventions and their synergistic combinations to optimize the facilitation of employees’ pro-environmental innovative behaviors.

## Data Availability

The raw data supporting the conclusions of this article will be made available by the authors without undue reservation.

## Footnotes

^1^Data source: www.stats.gov.cn/sj/ndsj/2023/indexch.htm.
