# Supplementary material for: How employees can break out of their learning comfort zone in green innovation scenarios: a nudging experiment based on the pressures of sustainable development in China
Source: Front Psychol. 2025 Jun 23;16:1430125. doi: 10.3389/fpsyg.2025.1430125 (PMC12232429; doi:10.3389/fpsyg.2025.1430125)
Supplement: Supplementary file 2 [file Presentation_1.pdf]

Appendix: questionnaire text

1. Control group

Hello, my dear friend. As you know, mankind is facing the common challenges of resource waste, environmental pollution, and destruction of biodiversity. To some extent, green innovation is conformed to be able to balance economic, social, and ecological benefits in the development of human society.

We expect to know about your interest in green innovation. Please be assured that your information will not be disclosed and will only be used for research purposes. Thank you very much for your time. Your reply is very important to us.

Green innovation place on applying new technology, process, institution, and management in your work. These actions aimed to reducing pollution and energy consumption, as well as increasing resources utilization and economic benefit. Green innovation may be increasing production costs in the initial stage. But after our efforts, green innovation can promote operation revenue, corporate reputation, environmental cleaning, and your profession competence.

We are known that green innovation is based on green knowledge. Let's find out how interested you are in green knowledge (Judge for yourself based on your work). Are you willing to put in the extra time to learn about green knowledge and stick with it? Please write down how many hours per week you study green knowledge in your free time, within a range of 0-10 hours (If you are prepared for more than 10 hours, please tell me the number as well). In addition, please select (1) your staff position from manager or non-manager, and (2) your enterprise type from state-owned enterprise or private enterprise.

2. Social norm group

Hello, my dear friend. As you know, mankind is facing the common challenges of resource waste, environmental pollution, and destruction of biodiversity. To some extent, green innovation is conformed to be able to balance economic, social, and ecological benefits in the development of human society.

According to our previous survey, 95% of employees in your industry agree that green innovation is important. Most employees are willing to put in the extra time to learn about green knowledge, and they expect you to do the same.

We expect to know about your interest in green innovation. Please be assured that your information will not be disclosed and will only be used for research purposes. Thank you very much for your time. Your reply is very important to us.

Green innovation place on applying new technology, process, institution, and management in your work. These actions aimed to reducing pollution and energy consumption, as well as increasing resources utilization and economic benefit. Green innovation may be increasing production costs in the initial stage. But after our efforts, green innovation can promote operation revenue, corporate reputation, environmental cleaning, and your profession competence.

We are known that green innovation is based on green knowledge. Let's find out how interested you are in green knowledge (Judge for yourself based on your work). Are you willing to put in the extra time to learn about green knowledge and stick with it? Please write down how many hours per week you study green knowledge in your free time, within a range of 0-10 hours (If you are prepared for more

than 10 hours, please tell me the number as well). In addition, please select (1) your staff position from manager or non-manager, and (2) your enterprise type from state-owned enterprise or private enterprise.

3. Social status group

Hello, my dear friend. As you know, mankind is facing the common challenges of resource waste, environmental pollution, and destruction of biodiversity. To some extent, green innovation is conformed to be able to balance economic, social, and ecological benefits in the development of human society.

We expect to know about your interest in green innovation. Please be assured that your information will not be disclosed and will only be used for research purposes. Thank you very much for your time. Your reply is very important to us.

Green innovation place on applying new technology, process, institution, and management in your work. These actions aimed to reducing pollution and energy consumption, as well as increasing resources utilization and economic benefit. Green innovation may be increasing production costs in the initial stage. But after our efforts, green innovation can promote operation revenue, corporate reputation, environmental cleaning, and your profession competence.

If you can participate in green innovation right now, your valuable exploration experience can provide guidance to your peers. Hence, your industry popularity and status may increase. More excitingly, seemingly small changes in your work can unleash great influence. Your behavior can change the people around you and the world for the better.

We are known that green innovation is based on green knowledge. Let's find out how interested you are in green knowledge (Judge for yourself based on your work). Are you willing to put in the extra time to learn about green knowledge and stick with it? Please write down how many hours per week you study green knowledge in your free time, within a range of 0-10 hours (If you are prepared for more than 10 hours, please tell me the number as well). In addition, please select (1) your staff position from manager or non-manager, and (2) your enterprise type from state-owned enterprise or private enterprise.

4. "Norm + Status" group

Hello, my dear friend. As you know, mankind is facing the common challenges of resource waste, environmental pollution, and destruction of biodiversity. To some extent, green innovation is conformed to be able to balance economic, social, and ecological benefits in the development of human society.

According to our previous survey, 95% of employees in your industry agree that green innovation is important. Most employees are willing to put in the extra time to learn about green knowledge, and they expect you to do the same.

We expect to know about your interest in green innovation. Please be assured that your information will not be disclosed and will only be used for research purposes. Thank you very much for your time. Your reply is very important to us.

Green innovation place on applying new technology, process, institution, and management in your work. These actions aimed to reducing pollution and energy consumption, as well as increasing resources utilization and economic benefit. Green innovation may be increasing production costs in the initial stage. But after our efforts, green innovation can promote operation revenue, corporate reputation, environmental cleaning, and your profession competence.

If you can participate in green innovation right now, your valuable exploration experience can provide guidance to your peers. Hence, your industry popularity and status may increase. More excitingly, seemingly small changes in your work can unleash great influence. Your behavior can change the people around you and the world for the better.

We are known that green innovation is based on green knowledge. Let's find out how interested you are in green knowledge (Judge for yourself based on your work). Are you willing to put in the extra time to learn about green knowledge and stick with it? Please write down how many hours per week you study green knowledge in your free time, within a range of 0-10 hours (If you are prepared for more than 10 hours, please tell me the number as well). In addition, please select (1) your staff position from manager or non-manager, and (2) your enterprise type from state-owned enterprise or private enterprise.

5. "Status + Norm" group

Hello, my dear friend. As you know, mankind is facing the common challenges of resource waste, environmental pollution, and destruction of biodiversity. To some extent, green innovation is conformed to be able to balance economic, social, and ecological benefits in the development of human society.

If you can participate in green innovation right now, your valuable exploration experience can provide guidance to your peers. Hence, your industry popularity and status may increase. More excitingly, seemingly small changes in your work can unleash great influence. Your behavior can change the people around you and the world for the better.

We expect to know about your interest in green innovation. Please be assured that your information will not be disclosed and will only be used for research purposes. Thank you very much for your time. Your reply is very important to us.

Green innovation place on applying new technology, process, institution, and management in your work. These actions aimed to reducing pollution and energy consumption, as well as increasing resources utilization and economic benefit. Green innovation may be increasing production costs in the initial stage. But after our efforts, green innovation can promote operation revenue, corporate reputation, environmental cleaning, and your profession competence.

According to our previous survey, 95% of employees in your industry agree that green innovation is important. Most employees are willing to put in the extra time to learn about green knowledge, and they expect you to do the same.

We are known that green innovation is based on green knowledge. Let's find out how interested you are in green knowledge (Judge for yourself based on your work). Are you willing to put in the extra time to learn about green knowledge and stick with it? Please write down how many hours per week you study green knowledge in your free time, within a range of 0-10 hours (If you are prepared for more than 10 hours, please tell me the number as well). In addition, please select (1) your staff position from manager or non-manager, and (2) your enterprise type from state-owned enterprise or private enterprise.
